# Acute Hemorrhagic Edema of Infancy With Targetoid Purpura and Mild Mucosal Involvement

**DOI:** 10.7759/cureus.100998

**Published:** 2026-01-07

**Authors:** Iman H Al-Rusheidi, Amany A Mansour

**Affiliations:** 1 Department of Dermatology, Al Musanaah Extended Health Center, Al Musanaah, OMN; 2 Department of Pediatrics, Al Musanaah Extended Health Center, Al Musanaah, OMN

**Keywords:** acute hemorrhagic edema of infancy, mucosal involvement, pediatric dermatology, purpura, targetoid lesions, vasculitis

## Abstract

Acute hemorrhagic edema of infancy (AHEI) is a rare leukocytoclastic vasculitis characterized by the abrupt onset of purpuric and targetoid lesions with edema in young children. A 13-month-old previously healthy girl presented with a rapidly progressive rash involving the face, trunk, and extremities following a brief febrile illness. Cutaneous examination revealed sharply demarcated purpuric papules and plaques with targetoid configuration and central duskiness, accompanied by mild lip erosions and shallow oral ulceration. The patient remained systemically well, and laboratory investigations were reassuring, with no evidence of renal, gastrointestinal, or hematologic involvement. Based on the characteristic clinical presentation and benign course, a diagnosis of AHEI was made, and a skin biopsy was not pursued. The patient was managed conservatively with supportive care. At one-week follow-up, the mucosal lesions had resolved completely, and the purpuric eruption had markedly improved. This case illustrates a classic presentation of AHEI with mild mucosal involvement and highlights key clinical features that aid in distinguishing it from other targetoid eruptions, allowing avoidance of unnecessary investigations and reassurance of caregivers.

## Introduction

Purpuric eruptions in infants often prompt urgent evaluation because of concern for serious infectious, hematologic, or vasculitic conditions. Acute hemorrhagic edema of infancy (AHEI) is an uncommon but distinctive leukocytoclastic vasculitis that typically affects children between four and 24 months of age and is characterized by the sudden appearance of purpuric plaques, targetoid lesions, and localized edema, most commonly involving the face and extremities [1,2].

Despite its alarming clinical appearance, AHEI follows a benign, self-limited course, with spontaneous resolution usually occurring within one to three weeks [1,3]. Antecedent respiratory or gastrointestinal infections, medications, and vaccinations have frequently been reported [1,3,4]. Systemic involvement is rare, which helps distinguish AHEI from Henoch-Schönlein purpura, which more commonly presents with renal, gastrointestinal, or joint manifestations [5].

Although mucosae are usually spared, lips or oral mucosa may rarely be involved, complicating the differential diagnosis. This may raise concern for erythema multiforme, Stevens-Johnson syndrome, or Mycoplasma-associated mucositis [6,7]. We report a classic case of AHEI with mild mucosal involvement and discuss key features that support clinical diagnosis without the need for biopsy.

## Case presentation

A 13-month-old previously healthy girl presented with a four-day history of a rapidly progressive rash involving the face, trunk, and extremities. The eruption was preceded by a low-grade fever and mild upper respiratory symptoms one day earlier. There was no recent medication use, vaccination, or travel history. The rash was painless and non-pruritic.

**Figure 1:**
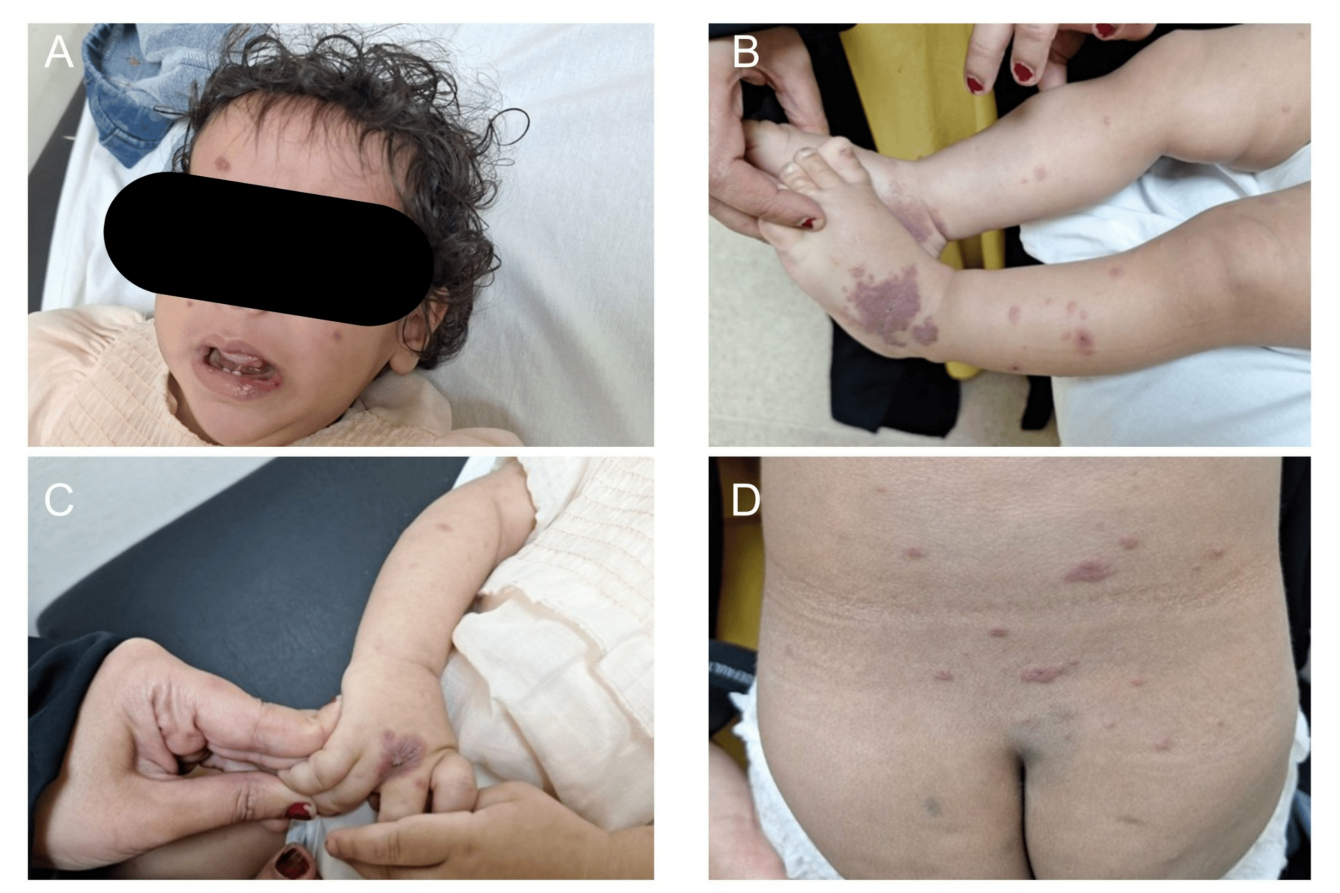
Initial presentation. Panels A–D demonstrate the acute onset of sharply demarcated purpuric and targetoid lesions with central duskiness and associated edema. Panel A shows facial involvement with purpuric plaques and mild lip erosions. Panels B and C illustrate purpuric plaques and edema involving the extremities. Panel D shows scattered purpuric lesions involving the trunk.

On examination, the child was alert, afebrile, and with no distress. Numerous sharply demarcated purpuric papules and plaques, some with targetoid configuration and central duskiness, were noted on the face, trunk, and extremities (Figure 1A-1D). There was no dermographism, and the lesions were non-blanchable. The lesions were fixed in location and did not demonstrate a migratory pattern. Mild superficial lip erosions and shallow oral ulceration were present (Figure 1A). The upper and lower extremities showed purpuric plaques with associated edema (Figure 1B, 1C), while scattered purpuric lesions were also observed on the trunk (Figure 1D). The palms, soles, and genital region were spared, and no grouped vesicles or bullae were identified. The remainder of the systemic examination was unremarkable.

Laboratory evaluation revealed mild leukocytosis with lymphocyte predominance and elevated C-reactive protein, while platelet count, coagulation profile, liver and renal function tests, peripheral blood smear, and urinalysis were within normal limits (Table 1).

**Table 1:** Laboratory investigations at initial presentation Reference ranges are based on the hospital laboratory’s age-appropriate internal reference values.

Category	Parameter	Result	Reference range
Complete blood count	White blood cell count	15.1 ×10³/µL	4.5–14.5 ×10³/µL
Complete blood count	Neutrophils	34.8% (5.2 ×10³/µL)	Not applicable
Complete blood count	Lymphocytes	56.2% (8.5 ×10³/µL)	Not applicable
Complete blood count	Red blood cell count	4.06 ×10⁶/µL	Not applicable
Complete blood count	Hemoglobin	11.0 g/dL	10.5–13.5 g/dL
Complete blood count	Hematocrit	33.1%	33–39%
Complete blood count	Mean corpuscular volume	81.5 fL	70–86 fL
Complete blood count	Mean corpuscular hemoglobin	27.1 pg	23–31 pg
Complete blood count	Mean corpuscular hemoglobin concentration	33.2 g/dL	30–36 g/dL
Complete blood count	Red cell distribution width	15.7%	11.5–16.5%
Complete blood count	Platelet count	202 ×10³/µL	150–450 ×10³/µL
Complete blood count	Mean platelet volume	9.1 fL	7–10.5 fL
Coagulation	Prothrombin time	9.8 s	10.3–12.5 s
Coagulation	International normalized ratio	0.84	0.9–1.1
Coagulation	Activated partial thromboplastin time	25.2 s	25.1–36.5 s
Inflammatory marker	C-reactive protein	36.38 mg/L	0–5 mg/L
Liver function	Alanine aminotransferase	9.94 IU/L	0–33 IU/L
Liver function	Alkaline phosphatase	142.9 IU/L	90–216 IU/L
Liver function	Total bilirubin	7.34 µmol/L	0–20 µmol/L
Liver function	Total protein	69.35 g/L	57–82 g/L
Liver function	Albumin	43.87 g/L	35–50 g/L
Renal function	Urea	1.74 mmol/L	1.8–6 mmol/L
Renal function	Creatinine	20.72 µmol/L	30–55 µmol/L
Electrolytes	Sodium	138.9 mmol/L	135–145 mmol/L
Electrolytes	Potassium	4.63 mmol/L	3.5–5.0 mmol/L
Electrolytes	Chloride	104.2 mmol/L	98–107 mmol/L
Blood film	Peripheral blood smear	Normocytic normochromic red cells; no polychromasia, nucleated red blood cells, or hemolytic features; normal white blood cell and platelet morphology	Not applicable
Urinalysis	White blood cells	Nil	Not applicable
Urinalysis	Red blood cells	Nil	Not applicable
Urinalysis	Nitrite	Negative	Not applicable
Urinalysis	Leukocyte esterase	Negative	Not applicable
Urinalysis	Protein	Negative	Not applicable
Urinalysis	Glucose	Negative	Not applicable
Urinalysis	Ketones	Negative	Not applicable
Urinalysis	Blood	Negative	Not applicable

The mildly elevated C-reactive protein was consistent with an acute inflammatory process. A normal platelet count and normal coagulation studies excluded thrombocytopenic and coagulopathic causes of purpura, including severe infectious etiologies such as meningococcemia. Normal renal function and urinalysis argued against Henoch-Schönlein purpura, while normal liver function tests and a normal peripheral blood film without hemolytic or malignant features supported the absence of systemic or hematologic disease.

Given the characteristic clinical presentation, absence of systemic involvement, reassuring laboratory findings, and early spontaneous improvement, the patient was provisionally diagnosed with AHEI, and a skin biopsy was not pursued. The patient was managed conservatively with oral antihistamines and topical corticosteroids.

**Figure 2:**
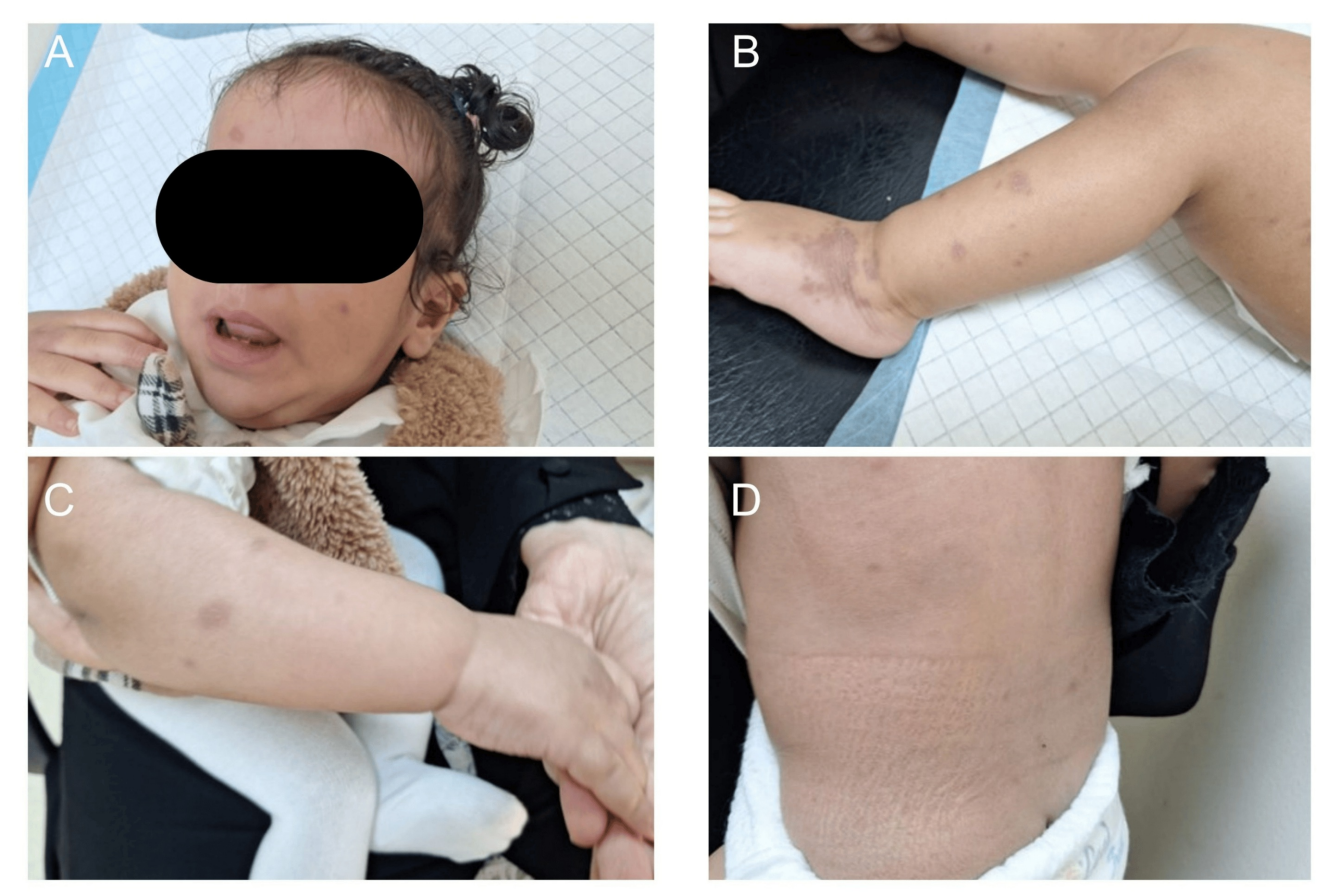
One-week follow-up. Panels A–D show marked clinical improvement one week after onset, with significant fading of the purpuric and targetoid lesions and resolution of associated edema. Facial lesions and prior lip erosions have resolved (Panel A), extremity lesions have faded with mild residual hyperpigmentation (Panels B and C), and truncal lesions have nearly completely resolved (Panel D).

At one-week follow-up, the patient showed marked clinical improvement. The facial lesions and lip erosions had resolved (Figure 2A), and the purpuric lesions on the extremities had significantly faded with resolution of edema, leaving mild residual hyperpigmentation (Figure 2B, 2C). Truncal lesions had nearly completely resolved (Figure 2D).

Written informed consent was obtained from the child’s legal guardian for treatment and for open-access publication, including the use of all clinical images.

## Discussion

AHEI is a distinctive form of leukocytoclastic vasculitis confined almost exclusively to infancy. The sudden onset of purpuric, often targetoid lesions accompanied by edema can appear dramatic but is typically benign and self-resolving [1-3]. Recognition of this condition is essential to prevent unnecessary investigations and caregiver anxiety.

Differentiation from other targetoid eruptions is critical. Henoch-Schönlein purpura may appear similar but is usually associated with abdominal pain, arthralgia, or renal involvement, none of which were present in this case [5]. Erythema multiforme is characterized by true three-zone target lesions, often evolving from vesicles or papules, and is more commonly associated with herpes simplex infection; it is also relatively uncommon in early infancy [6]. Urticaria multiforme was also considered, as it may present in young children with atypical targetoid lesions and a benign course. However, in our patient, the lesions were fixed, purpuric, non-blanchable, painless, and non-pruritic, with no dermographism and no migratory pattern, favoring a diagnosis of AHEI.

More severe conditions, such as Stevens-Johnson syndrome and Mycoplasma-associated mucositis, typically involve extensive mucosal disease and systemic symptoms, which were absent in this patient [6,7]. Invasive bacterial infections, including meningococcemia, were excluded based on the child’s good appearance, stable vital signs, and normal laboratory investigations [8]. Kawasaki disease was also unlikely given the lack of persistent fever and characteristic mucocutaneous features [9].

Although histopathology demonstrates leukocytoclastic vasculitis, multiple reports emphasize that biopsy is unnecessary when the clinical presentation is typical and the child remains systemically well [3,4,10]. The rapid improvement documented at one week, with resolution of edema and mild residual hyperpigmentation, further supports the benign and self-limited nature of AHEI, even in the presence of mild mucosal involvement. Recurrence is rare, and long-term sequelae have not been reported.

## Conclusions

AHEI should be considered in infants presenting with sudden purpuric or targetoid lesions and localized edema, particularly when systemic findings are absent, and laboratory studies are unremarkable. Mild mucosal involvement does not exclude the diagnosis. Accurate clinical recognition allows avoidance of invasive procedures and reassurance of caregivers, as the prognosis is excellent with supportive care alone.
